# Ecological dynamics of plasmid transfer and persistence in microbial communities

**DOI:** 10.1016/j.mib.2022.102152

**Published:** 2022-08

**Authors:** Michael J Bottery

**Affiliations:** Division of Evolution Infection and Genomics, School of Biological Sciences, University of Manchester, Manchester M13 9PL, UK

## Abstract

Plasmids are a major driver of horizontal gene transfer in prokaryotes, allowing the sharing of ecologically important accessory traits between distantly related bacterial taxa. Within microbial communities, interspecies transfer of conjugative plasmids can rapidly drive the generation genomic innovation and diversification. Recent studies are starting to shed light on how the microbial community context, that is, the bacterial diversity together with interspecies interactions that occur within a community, can alter the dynamics of conjugative plasmid transfer and persistence. Here, I summarise the latest research exploring how community ecology can both facilitate and impose barriers to the spread of conjugative plasmids within complex microbial communities. Ultimately, the fate of plasmids within communities is unlikely to be determined by any one individual host, rather it will depend on the interacting factors imposed by the community in which it is embedded.

**Current Opinion in Microbiology** 2022, **68**:102152

This review comes from a themed issue on **Environmental Microbiology**

Edited by **Paul Hoskisson** and **John BruceI**

For complete overview of the section, please refer to the article collection, “Environmental Microbiology”

Available online 2nd May 2022

https://doi.org/10.1016/j.mib.2022.102152

1369-5274/© 2022 The Author(s). Published by Elsevier Ltd. This is an open access article under the CC BY license (http://creativecommons.org/licenses/by/4.0/).

## Introduction

Horizontal gene transfer (HGT) occurs extensively among prokaryotes and is a major driver of bacterial evolution and diversification, with large proportions of bacterial genomes consisting of horizontally acquired genes [1]. Mobile genetic elements, such as plasmids, phages, and integrative conjugative elements, act as vectors of HGT, enabling the sharing of genes between distantly related bacteria 2, 3, 4. Plasmids are major drivers of HGT within bacterial communities, encoding core genes required for their own independent replication and, in the case of conjugative plasmids, their transfer between hosts. Importantly, in addition to core genes, plasmids encode accessory genes that are not critical for their own propagation, but rather provide ecologically contingent selective advantages to their hosts. Such beneficial accessory genes can accelerate adaption to novel or fluctuating environments by providing ‘plug and play’ traits, bypassing the requirement for rare *de novo* mutations 4, 5. The incredible impact of plasmid transfer upon bacterial evolution is illustrated by the rapid spread of antimicrobial resistance in response to the anthropogenic use of antibiotics.

Modern advances in sequencing technologies have unearthed a huge diversity of plasmids within neutral microbial communities 6, 7, 8, 9; however, the dynamics of conjugative plasmids within communities have received relatively little attention. Within single-species populations, plasmid dynamics are considered to be governed by a combination of factors specific to the host–plasmid paring [10], including rates of horizontal transfer [11], rates of lost through imperfect segregation, positive selection for accessory traits, and purifying selection due to fitness costs imposed on their hosts [12]. However, in natural environments, plasmid dynamics are unlikely to occur solely in isolated single-species populations. Rather, plasmids will be embedded within a wider community context which not only has the potential to alter the relative impact of each of these intrinsic host–plasmid properties but also imposes additional factors which can alter the ecological dynamics of plasmid persistence and transfer within communities.

Here, I summarise the latest advances in our understanding of how community context, that is, species diversity together with interspecies interactions, can impact the dynamics of conjugative plasmids by facilitating or limiting plasmid transmission in natural microbial communities. This perspective focuses predominately on the persistence and spread of conjugative plasmids within communities. While the factors altering the stability of non-mobilisable plasmids may be different, due to interspecies and multitrophic interactions potentially altering host–plasmid properties (such as fitness costs), it remains likely that community context will also impact the persistence of non-mobilisable plasmids.

## Transfer of conjugative plasmids within multispecies microbial communities is common

Diverse multispecies microbial communities are considered hotspots for HGT; in such communities, conjugative plasmids are capable of rapidly spreading between diverse taxa and persisting for long periods of time 6, 13, 14. The presence of plasmids can dramatically alter the ecology of natural communities, for example, by providing resistance to heavy metals [15], encoding genes essential for virulence [16], or facilitating the spread of antibiotic resistance 17, 18••. Conjugative plasmids, for example, are instrumental in shuttling transposons harbouring carbapenem resistance genes between *Enterobacterales*, causing multispecies outbreaks of carbapenem-resistant infections 19, 20, 21, 22. A retrospective analysis of within-patient dynamics of carbapenemase-encoding pOXA-48 plasmids showed that transfer among members of the gut microbiota is rife, and is likely responsible for the long-term establishment of antibiotic resistance within patients [18]. Tracking the temporal dynamics of natural plasmids *in situ* within infant gut microbiota has also shone a light on the importance of conjugative plasmid transfer in the spread of virulence factors and antibiotic resistance genes between coexisting bacterial lineages [23], even in the absence of antibiotic selection [24]. In addition to host-associated microbiomes, conjugative plasmid are important for the survival and persistence of multidrug resistance in microbial communities of wastewater treatment plants (WWTP) [25], with WWTPs proposed to act as revisors for the spread of antibiotic resistance plasmids to other natural communities such as soil communities [26].

The host range of a conjugative plasmid is a key factor determining its ability to spread to a diverse subset of a community. While some plasmids are restricted to a single or closely related subset of species due to limitations in the ability to form mating pairs or incompatibility between the plasmid replication system and their hosts [27], other plasmids have a remarkable board host range, often being able to transfer between different genera or phyla. Studies tracking conjugation through fluorescence-activated cell sorting in both natural and synthetic communities containing fluorescently labelled broad host-range plasmids have demonstrated the ability of plasmids to spread to multiple diverse taxa within short periods of time 28, 29, 30. When introduced to mice gut microbiomes, plasmids containing the RP4 conjugation system — capable of conjugating to both Gram-negative and Gram-positive bacteria — were able to rapidly spread throughout the community even when the original plasmid donor was unable to colonise the gut [30]. Similar results were observed in both WWTP [29] and soil [28] communities, where broad host-range plasmids were able to invade a phylogenetically diverse subset of the community. While the host range is a key factor determining the ability of a plasmid to invade diverse members of a microbiome [27], questions remain on how the specific ecological properties of a community influence the successful maintenance and spread of plasmids.

## Factors limiting plasmid transfer and persistence within microbial communities

**Figure 1:**
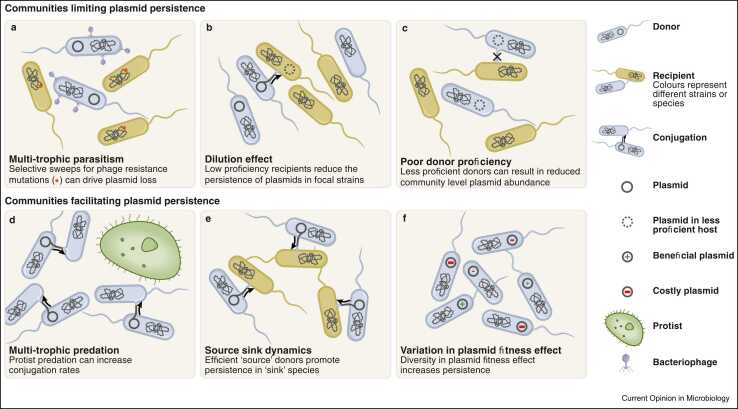
Summary of the discussed factors within multispecies communities that can act to limit **(a**–**c)** or facilitate **(d**–**f)** the persistence of plasmids. **(a)** Parasitism, for example, by bacteriophage, can drive selective sweeps for resistance mutations resulting in the loss of plasmids. **(b)** The presence of less permissive hosts in a community can dilute the transfer efficiency of conjugative plasmids reducing the stability of plasmids that rely on infectious transfer for their maintenance. **(c)** The proficiency of the original donor determines the ability of a plasmid to become established within a community, with less proficient donors being unable to disseminate plasmids to a wide diversity of novel hosts. **(d)** Protist predation can maintain populations in continuous growth phase which elevates conjugation rates and increases plasmid persistence. **(e)** Highly efficient donors can act as sources of plasmids, maintaining them in less proficient hosts due to high intraspecies conjugation rates. **(f)** Variation in plasmid fitness effects can stabilise plasmids within populations even when the net effect of harbouring a plasmid is costly.

Microbial communities are rightly considered hotspots for HGT; however, barriers to plasmid-mediated HGT exist both at the single cell level (reviewed in Ref. [27]) and imposed by the ecology of the wider community. Multitrophic interactions, unequal plasmid persistence between different hosts, and genomic diversity of microbial communities can limit both the establishment and propagation of conjugative plasmids within communities (Figure 1a-c). However, the ecology of a community is unlikely to preclude plasmids from a community, rather it may limit the ability of specific plasmids to spread within a subset of a community.

### Donor proficiency

The stability [31], fitness effects 32, 33, and conjugation rates 11, 34, 35, 36 of plasmids can differ between even closely related bacteria. Such differences in host proficiency can have major impacts on the ability of a donor to disseminate conjugative plasmids within communities [37]. Whereas highly proficient host strains have been shown to stably establish plasmids in novel communities [28], the same plasmid in less proficient donor strains may fail to spread within a community. For example, Heß et al. [38] demonstrated how the spread of the broad host-range plasmid RP4 within a synthetic 21 species community depended upon the initial donor strain. While specific *Escherichia coli* donor strains facilitated the stable maintenance of the antibiotic resistance plasmid in the community, other *E. coli* donor strains were unable to stably establish the plasmid within the community. Importantly, differences between the donors’ ability to disseminate the plasmid were not due to differential impacts of the invading strain upon the community composition, but rather due to reduced stability of the plasmid within the less proficient host strain. Similarly, the proficiency of the original donor determined the ability of a large conjugative mercury resistance plasmid, pQBR103, to spread within a multispecies community [39], with less proficient hosts resulting in a lower community-level abundance of the conjugative plasmid. The impact of host proficiency is also reflected in the spread of clinical extended-spectrum beta-lactamase (ESBL)-producing plasmids in *in vivo* mice gut communities, where Benz et al. [40] showed that donor, plasmid and recipient identities all contributed to the frequency of transconjugants within microbial communities.

### Diversity limiting infectious transfer

Highly conjugative plasmids may be able to maintain sufficient rates of infectious transmission to persist within microbial communities despite imposing high fitness costs upon their hosts [41]. Recent studies have identified multiple conjugative plasmids with sufficiently high conjugation rates to persist purely as infectious elements in the absence of positive selection 11, 42. However, the maintenance of costly plasmids purely by infectious transmission is likely to be unstable in the face of even minor perturbations to conjugation rate. Diverse multispecies communities will contain a diversity of possible hosts, each of which will vary in their ability to harbour and spread conjugative plasmids [43]; less-permissive hosts with low conjugation rates will introduce barriers to the infectious spread of conjugative plasmids within the community and may ultimately destabilise the maintenance of purely infectious plasmids. An elegant study by Kottara et al. [44] demonstrated this effect within communities, establishing that the dilution effect — a hypothesis in disease ecology [45] whereby disease risk is reduced as a result of less efficient disease vectors diluting the impact of highly competent vectors — can equally apply to the infectious spread of plasmids. Through tracking the spread of a highly conjugative plasmid, pQBR57, within multispecies communities, it was shown that the presence of a less permissive host with significantly lower conjugation rates reduced the overall infectivity of the plasmid within the focal species [44]. Thus, within bacterial communities, the spread of plasmids that rely purely upon infectious transmission for their maintenance may be constrained by the properties of the communities’ members. Community composition may therefore impact the types of plasmids that are harboured within them, preferentially selecting plasmids that do not purely rely on infectious transfer, but rather supporting plasmids that are stably maintained within multiple hosts.

The diversity of host defences against foreign DNA present within a microbial community is also likely to play an important role in the ability of infectious plasmids to spread within communities 43, 46, creating barriers to transfer into subsets of the community. It has been shown that restriction–modification systems can promote the preferential transfer of conjugative plasmids to kin in naturally co-occurring *E. coli*, and that CRISPR-Cas immunity targets plasmids more often than phage within microbial communities in activated sludge biological WWTP [46]. Although it has been proposed that positive selection for plasmid-encoded traits should result in selection against host immunity, and immunity to costly plasmids should be under positive selection [47], how diversity in host immunity impacts the dynamics of plasmid transfer within natural communities remains uncertain.

### Multitrophic interactions

Interactions between trophic levels, including parasitism from bacteriophages and predation from protists, can affect the ecological structure of a community and impose strong evolutionary selective pressures upon a communities’ members 48, 49, 50. Such effects upon communities can in turn impact plasmid dynamics. Parasitism by phage, for example, can limit plasmid persistence within bacterial populations due to the combined effect of the ecological impacts of increased mortality and the evolutionary consequences of phage resistance. Harrison et al. [51] showed recurrent selective sweeps of phage resistance mutations can initially stabilise costly plasmids, but ultimately drove the loss of plasmids from populations of *Pseudomonas fluorescens* [51]. Similar results have been observed during prophage infection, where plasmid spread is limited due to an increased mortality rate in the plasmid carrying fraction of the population [52]. Some phages can also specifically target the conjugation machinery of plasmids, selecting against plasmid carriage resulting in the loss of the plasmid and hence the phage receptor, as a mechanism of phage resistance [53]. To what extent bacteriophage limit the spread of plasmids within multihost, multiphage communities remains unexplored. For example, within multispecies bacterial communities, it may be possible for broad host-range plasmids to find refuge in bacterial species that are outside the host range of the phage.

As with bacteriophage parasitism, the impact of protozoan predation upon plasmid persistence within bacterial populations is dependent upon both the ecological and evolutionary effects of increased mortality. Predation by *Tetrahymena thermophila* resulted in the loss of non-conjugative plasmids from *Serratia marcescens* populations due to interactions between the evolution of costly grazing resistance and the cost of plasmid carriage [54]. However, the ecological effects of reduced population density had the opposite effect upon conjugative plasmids; protist predation maintained bacterial populations in a constant growth phase resulting in elevated conjugations rates, increasing the stability of the plasmid via infectious transfer [54]. However, within bacterial communities protist predation can alter the ecological structure and diversity of a community, subsequently affecting the dynamics of conjugative plasmid transfer [55]. Carins et al. [55] demonstrated how the identity of transconjugants within a 62-strain community significantly differed in the presence and absence of protist predation. As grazing affected community diversity, the ability of the plasmid to spread to specific members of the community was limited.

The interacting effect of bacteriophage and protists upon plasmid dynamics has also been explored by Carins et al. [56] within three-way communities consisting of *E. coli* harbouring the RP4 conjugative plasmid, the plasmid-dependent PRD1 phage and protist, *T. thermophila*. Phage parasitism alone drove the conjugative plasmid extinct as previously observed [53]; however, in the presence of protist predation, the plasmid was maintained despite the purifying effect of the phage due to increased conjugation rates induced by the ecological effects of protist grazing. Oscillating selection pressures for and against conjugation and plasmids carriage induced by multitrophic interactions may play a critical role in plasmid transfer and persistence within natural bacterial communities.

## Community diversity as a facilitator of plasmid persistence

Recent studies have shown that the key traits that determine a plasmids persistence within a population (fitness cost, segregation rate, and conjugation rate) can vary for the same plasmid in different host backgrounds 57, 58. Thus, a diverse community that contains many possible hosts will likely encompass a wide diversity of abilities to stably maintain and disseminate plasmids. Although host diversity can introduce barriers to transmission in focal species, for example via the dilution effect, a diverse community may also contain highly permissive hosts that can act as plasmid reservoirs and promote community-level plasmid persistence (Figure 1d-f).

### Source-sink transfer dynamics

Conjugation rates can differ greatly between different genetic backgrounds [34], with some hosts being able to stably maintain plasmids purely through infectious transfer 11, 59. Such highly efficient donor strains may act as a plasmid ‘source’ within microbial communities, disseminating plasmids to less permissive ‘sink’ species, thus increasing the persistence of the plasmid across the community [59]. In experimental soil communities, Hall et al. [59] showed that the mercury resistance plasmid pQBR57 could be maintained within single species populations of *P. fluorescence* through conjugation while imposing high fitness costs, whereas the same plasmid was lost from monoculture *Pseudomonas putida* populations, despite imposing a lower fitness burden, due to significantly lower intraspecies conjugation rates. However, in coculture, *P. fluorescence* was able to maintain pQBR57 in less the permissive *P. putida* at low frequency due to interspecies conjugation. Interestingly, positive selection for mercury resistance led to the mercy resistance gene being transferred to the chromosome of *P. putida* in single-species populations. Subsequently, the plasmid was lost from these population, being replaced by chromosomal resistance mutants. Although this alternative route to mercury resistance clearly benefited *P. putida* in single-species populations, during mercury treatment of coculture populations chromosomal resistant mutants were less common and source-sink transfer maintained the plasmid within *P. putida*. Within more complex multispecies mouse gut communities, Ronda et al. [30] demonstrated that novel conjugative plasmids were only able to persist in the native gut microbiome when the original donor was able to colonise the established community. Although non-gut-adapted donors initially transferred the plasmid to a wide range of novel hosts, the plasmid was unstable in the established gut community and was rapidly lost following the loss of the donor, which was unable to colonise the gut. In contrast, when a plasmid permissive native member of the gut was used that could effectively colonised the entirety of the gut, transconjugants in the microbiome were detectible throughout the experiment. Here, the stability of the plasmid in the wider gut community was dependent upon the continuous presence of the plasmid permissive source.

### Diversity in fitness effects enabling community persistence

Plasmids often cause physiological changes to their hosts that reduce the competitive fitness plasmid bearing bacteria [12]. Such fitness costs are a key determinant in the existence conditions of plasmids within single-species bacterial populations and can drive the adaptation of both the host and plasmid through the selection of compensatory mutations that ameliorated the costs of plasmid carriage 60, 61, 62, 63. However, the fitness effects of plasmids can differ between species; such variability in fitness effects may aid the long-term community-level persistence of plasmids. Through monitoring the persistence of broad host-range IncP1 plasmid pKJK5 within complex activated sludge microbial communities, Li et al. [64] showed that the plasmid could persist even in the absence of conjugal transfer and selection. Mapping the fitness effects of plasmid carriage to each taxon present in the community revealed a very wide distribution of fitness effects. Plasmids were maintained within permissive phylotypes including *Acinetobacter*, *Pseudomonas*, and *Enterobacteriaceae*, in which plasmid carriage either incurred no cost, or was beneficial. However, the survival of the plasmid within the community was dependent upon the abiotic environment. Cultivation of the community in anoxic conditions and specific nutritional environments lead to the loss of the plasmid from the community as the permissive phylotypes were selected against, suggesting the fitness effects were dependent upon the environmental context.

A similar diversity of plasmid fitness effects was also observed in co-occurring enterobacteria strains isolated from the gut microbiota of hospital patients [65]. Alonso-del Valle et al. [65] found that the clinically isolated OXA-48 carbapenemase carrying plasmid pOXA-48_K8 produced on average a small reduction in fitness in the absence of antibiotic selection. However, the plasmid fitness effects varied among different host strains following a normal distribution. Plasmid carriage had a minimal effect on most enterobacteria strains tested; however, in a subset of strains, the plasmid incurred a significant cost, while others gained a significant benefit from plasmid carriage. Mathematical modelling of multistrain bacterial communities showed that such variation in plasmid fitness effects promoted the maintenance of, on average, costly plasmid within the community. In contrast, simulations with no variance in cost between members of the community rapidly led to plasmid extinction. Moreover, community-level stability intuitively increased as the number of strains within the communities increased, as there was a greater probability of diverse communities containing a permissive strain where plasmid carriage was neutral or beneficial. Importantly, increased host diversity and sufficient variance in fitness effect reduced the requirement of conjugal horizontal transfer for plasmid stability, with plasmid being able to persist even in the absence of conjugation reflecting the results of Li et al. [64]. Although the genetic basis of diversity in plasmid costs, and in particular fitness advantages, is unclear, permissive host strains or species could aid the community-level maintenance of plasmids by providing stable plasmid reservoirs.

## Concluding remarks

Our understanding of the dynamics of conjugative plasmids has historically focused on controlled relatively simple single-species *in vitro* populations. However, it is becoming increasingly evident that the plasmid dynamics within natural microbial communities are much more complex. Communities are likely to contain a high diversity of plasmid–host interactions, ranging from completely incompatible hosts to highly proficient hosts. However, the dynamics of conjugative plasmid transmission and persistence within a community will not be solely dependent upon any one host, rather it will dependent upon the ecology context in which the plasmid is set. Importantly, the ecological properties of a community that can facilitate the persistence of plasmids, can in different contexts also limit the spread of the plasmid within the community. For example, while increased diversity of host permissiveness can increase stability via spill over transmission from proficient reservoir donors, the presence of non-permissive hosts can also dilute and destabilise the spread of purely infectious plasmids. Multi-tropic interactions, such as predation and parasitism from protists and phage, can promote conjugation plasmids, but equally drive plasmids to extinction.

The relative importance of different variables and how they interact to facilitate or limit the spread of plasmids within natural communities, are major outstanding questions. The complexity of microbial communities will make it challenging to answer such questions, in part due to the diversity of hosts — differing in their ability to receive, maintain and disseminate plasmids — as well as the diversity of interspecies and multitrophic interactions that are present within natural communities. A combination of approaches including the use of more representative *in vitro* multispecies and multitrophic communities (such as 54, 56), increased focus on theoretically modelling the effects of diversity and interspecies interactions (such as 52, 65••), and observing the dynamics of plasmid transfer within natural microbiomes (such as [24]) will all contribute to developing a deeper understanding of plasmid dynamics in complex communities.

## Conflict of interest statement

The authors declare that they have no known competing financial interests or personal relationships that could have appeared to influence the work reported in this paper.
